# Youth and Age

**Published:** 1863-10

**Authors:** 


### YOUTH AND AGE.

*From the Etonian.*

I often think each tottering form
That limps along in life's decline,
Once bore a heart as young and warm,
As full of idle thoughts as mine!
And each has had its dream of joy,
Its own unequaled, pure romance,
Commencing when the blushing boy
First thrills at lovely woman's glance.

And each could tell his tale of youth,
Would think its scenes of love evince
More passion, more unearthly truth
Than any tale, before or since.
Yes! they could tell of tender lays,
At midnight penned in classic shades,
Of days more bright than modern days,
And maids more fair than modern maids:

Of whispers in a willing ear,
Of kisses on a blushing cheek,
Each kiss, each whisper far too dear,
For modern lips to give or speak:
Of prospects, too, untimely crossed,
Or passions slighted or betrayed:
Of kindred spirits early lost,
And buds that blossomed but to fade:

Of beaming eyes and tresses gay,
Elastic form and noble brow,
And charms that all have passed away,
And left them what we see them now.
And is it thus?—is human love
So very light and frail a thing?
And must youth's brightest visions move
Forever on time's restless wing?

Must all the eyes that still are bright,
And all the lips that talk of bliss,
And all the forms so fair to sight,
Hereafter only come to this?
Then what are earth's best visions worth,
If we at length must leave them thus?
If all we value on this earth,
Ere long must fade away from us?

If that one being whom we take
  From all the world, and still recur
To all she said, and for her sake
  Feel far more joy when far from her;
If that one form which we adore,
  From youth to age, in bliss or pain,
Soon withers and is seen no more—
  Why do we love—if love be vain?

